# Quantifying Digital Biomarkers for Well-Being: Stress, Anxiety, Positive and Negative Affect via Wearable Devices and Their Time-Based Predictions

**DOI:** 10.3390/s23218987

**Published:** 2023-11-05

**Authors:** Berrenur Saylam, Özlem Durmaz İncel

**Affiliations:** Computer Engineering Department, Boğaziçi University, 34342 İstanbul, Türkiye; ozlem.durmaz@bogazici.edu.tr

**Keywords:** deep learning, LSTM, regression, ensemble learning, random forest, XGBoost, wearable devices, well-being, digital health, pervasive health, digital biomarkers

## Abstract

Wearable devices have become ubiquitous, collecting rich temporal data that offers valuable insights into human activities, health monitoring, and behavior analysis. Leveraging these data, researchers have developed innovative approaches to classify and predict time-based patterns and events in human life. Time-based techniques allow the capture of intricate temporal dependencies, which is the nature of the data coming from wearable devices. This paper focuses on predicting well-being factors, such as stress, anxiety, and positive and negative affect, on the Tesserae dataset collected from office workers. We examine the performance of different methodologies, including deep-learning architectures, LSTM, ensemble techniques, Random Forest (RF), and XGBoost, and compare their performances for time-based and non-time-based versions. In time-based versions, we investigate the effect of previous records of well-being factors on the upcoming ones. The overall results show that time-based LSTM performs the best among conventional (non-time-based) RF, XGBoost, and LSTM. The performance even increases when we consider a more extended previous period, in this case, 3 past-days rather than 1 past-day to predict the next day. Furthermore, we explore the corresponding biomarkers for each well-being factor using feature ranking. The obtained rankings are compatible with the psychological literature. In this work, we validated them based on device measurements rather than subjective survey responses.

## 1. Introduction

Wearables, such as smartwatches, fitness trackers, and biosensors, have gained popularity due to their ability to continuously monitor various physiological signals and capture temporal patterns in real-time. This wealth of temporal data provides valuable insights into human activities, health monitoring, and behavior analysis and opens up new possibilities for personalized, context-aware applications that can continuously monitor and classify temporal patterns to support various domains, including healthcare, fitness, and productivity enhancement.

Human well-being monitoring and prediction [1] is one of the example application areas focusing on an individual’s overall state of physical, mental, and social health, reflecting their sense of contentment, happiness, and fulfilment in life. World Health Organization (WHO) defines a well-being index (WHO-5) [2] with 5 factors based on the answers given to the following questions: ‘I have felt cheerful and in a good spirit’, ‘Calm and relaxed’, ‘Active and vigorous’, ‘Woke up fresh and rested’, ‘Daily life interests me’. The answers range from ‘All of the time’ to ‘At no time’ with six possible inputs.

In recent years, there has been a growing interest in leveraging wearable devices to monitor and predict individuals’ well-being and mental health in the literature [3,4,5,6]. This trend has emerged as a response to the increasing awareness of the importance of mental health in modern society and the growing data size collected by wearables. Traditional machine-learning algorithms, such as logistic regression, decision trees, and ensemble methods, have been extensively utilized in classifying or predicting well-being using data collected from wearables and questionaries. However, the time aspect of the temporal data, in other words, how previous data impact future well-being levels, is often overlooked in related studies. Wearables offer a unique opportunity to collect longitudinal data, enabling a deeper understanding of how stress, anxiety, and emotional states fluctuate over time [7,8]. Time-based prediction techniques, when combined with wearable devices, can offer a unique opportunity to capture and analyze temporal patterns. This integrated approach can provide valuable insights into individuals’ emotional states over time, facilitating early detection and timely interventions for improved mental well-being.

In addition, deep-learning architectures have emerged as powerful tools for time-based prediction problems. Recurrent Neural Networks (RNNs) and convolutional neural networks (CNNs) have gained attraction in well-being research [9,10,11,12,13,14]. RNNs, particularly LSTM variants, have demonstrated their ability to capture long-term dependencies and temporal dynamics in well-being-related data, while CNNs extract hierarchical features from wearable sensor data, enabling more accurate prediction. Furthermore, the combination of deep-learning architectures with wearable devices has led to the development of hybrid models that leverage both wearable sensor data and contextual information. These models incorporate multimodal inputs, such as physiological signals, accelerometer/activity data, and contextual features like time of day, location, social interactions, and self-reported mood states. By fusing multiple data sources that may impact an individual’s well-being levels, the model’s performance can be improved.

As well as deep-learning algorithms, ensemble methods are also powerful tools. Ensemble models combine multiple base classifiers to make predictions, leveraging the strengths of different models and reducing the impact of individual model weaknesses. Techniques like Bagging, Boosting, and Random Forests have been successfully employed in time-based classification tasks, yielding improved performance and robustness.

In this study, specifically, we focus on workplace well-being prediction using the Tesserae dataset [15], which was collected over an extended period from office workers. The primary objective is to predict the stress, anxiety, and positive and negative affect levels by analyzing the impact of past measurements on their well-being. There is also the sleep factor in the definition of the well-being index based on the WHO-5 well-being questionnaire [2]. Although the sleep response was recorded in the dataset, once we applied the same preprocessing steps to compare each target value’s results fairly, we were left with a limited amount of data due to the lack of intersection in missing values in parameter space and target value, i.e., sleep which is collected three to four times in a week even though they are classified in daily surveys. Thus, we could not include this fifth factor in the study.

Given the fact that the dataset is a time stream, we conducted experiments using prediction algorithms, namely Random Forest (RF), XGBoost as example ensemble algorithms, and LSTM as an example deep-learning algorithm, in their conventional versions as well as in time-based versions. Time-based versions use the concept of time lags to consider the time aspect of the data. In order to create the time-based versions, each data point is associated with previous time steps (i.e., days) to incorporate historical information. We aimed to observe the impact of previous, such as daily, weekly, and longer periods, well-being factors on the upcoming ones. We focus on the following research questions (RQ) in our study:

- RQ1: What are the underlying factors (biomarkers) of well-being for workers considering four main parameters: stress, anxiety, positive and negative affect?
- RQ2: Are these biomarkers compatible with conventional psychological studies?
- RQ3: Do time-based versions of the conventional algorithms help to improve prediction performances?

The rest of the paper is organized as follows: Section 2 explores the literature related to wearables and well-being monitoring. In Section 3, we explain the details of the dataset, the data construction step for our analysis, the data preprocessing step before analysis, and details of the target values, i.e., stress, anxiety, positive and negative affect. In Section 4, we provide the obtained results with feature ranking, modality ranking, and performance details with an interpretation, discussion, and comparison to the related studies, and we finish with Section 5 as a conclusion.

## 2. Related Work

Well-being, a multidimensional concept encompassing various facets of emotional and psychological health, has become a focal point of research [1,5,16,17,18] and early health intervention by continuous monitoring [19,20]. Within the spectrum of well-being, stress, anxiety, positive affect, and negative affect play pivotal roles in determining an individual’s mental and emotional state [21,22]. Stress and anxiety can significantly impact one’s overall well-being, and understanding and managing these factors are essential for promoting mental health [21,23,24].

Studies have explored the integration of wearable sensors, such as heart rate monitors, electrodermal activity sensors, accelerometers, and even Bluetooth beacons, to capture physiological and behavioral signals associated with stress and emotional well-being [25,26]. These sensors provide non-intrusive and continuous data streams that offer insights into individuals’ emotional experiences and their positive and negative affect.

Nevertheless, the most studied factor is the stress in the literature [27,28,29,30,31,32,33]. In recent years, stress prediction and assessment have witnessed a surge in innovative research aimed at harnessing diverse data sources and cutting-edge machine-learning techniques. These efforts have collectively contributed to a deeper understanding of stress patterns across different contexts and data modalities, paving the way for more effective stress management strategies.

Investigating the feasibility of stress prediction based on behavioral data, researchers have delved into smartphone activity as a potential stress indicator [28]. Leveraging machine-learning algorithms, this study dissects smartphone behaviors to uncover stress-indicative patterns, therefore contributing to a deeper understanding of stress dynamics in the digital age. By focusing on digital behavior, the study presents a unique perspective on stress prediction, emphasizing the significance of smartphone interactions in capturing stress-related cues.

Wearable technology emerges as another avenue for stress prediction. Employing physiological sensors, studies have endeavored to predict and visualize work-related stress through wearable sensing [29]. Heart rate and skin conductance are monitored to develop models that provide real-time insights into stress levels, thus empowering individuals and organizations with tools for effective stress management. By focusing on physiological markers, this research offers a unique approach to stress prediction, emphasizing the importance of wearable technology in monitoring and mitigating stress.

Moreover, the analysis of physiological signals has yielded significant strides in stress detection under real-life conditions [30]. By scrutinizing heart rate variability and electrodermal activity, researchers provide valuable insights into physiological stress markers, enhancing our understanding of stress dynamics in natural environments. This study’s focus on physiological markers in real-life settings offers a nuanced perspective on stress detection, providing insights into stress responses beyond controlled environments.

Mobile sensing has also paved the way for predicting stressful life events by analyzing sensor data such as location and physical activity [31]. This novel approach enhances our comprehension of stress-inducing contexts and contributes to users’ overall well-being by identifying potential stress triggers. This approach shifts the focus from immediate stress levels to predicting stress-inducing situations, highlighting the potential of preemptive interventions.

Intriguingly, multimodal approaches have been explored by combining speech and wearable sensor data for stress detection [32]. By integrating speech features and physiological signals, researchers have showcased the potential of using multiple data sources to achieve more accurate stress prediction models. The study’s emphasis on combining speech and physiological data offers a comprehensive approach to stress detection, leveraging multiple modalities to enhance prediction outcomes.

Comprehensive stress and sleep prediction strategies have incorporated physiological signals and smartphone data [33,34]. The fusion of heart rate variability, accelerometer data, and self-reported stress levels yields enhanced accuracy, demonstrating the potential of amalgamating diverse data sources for holistic stress assessment. This research stands out for its comprehensive integration of physiological signals and smartphone data, offering a multifaceted perspective on stress prediction [33]. In [34], the approach of combining wearables and phone sensors enables more accurate sleep detection by leveraging the benefits of both streams: combining wearable movement detection with mobile phone technology usage detection is employed. They showed that the combination of phone activity and wearables might produce better models of self-reported sleep than either stream alone on the Tesserae dataset.

There are also recent studies focusing on monitoring and predicting stress levels in the workplace. In a recent study [27], researchers developed a mobile app to collect a comprehensive dataset from 30 workers over 8 weeks. The app prompted users to complete a questionnaire three times daily, assessing stress, sleep quality, work abandonment, energy levels, and mood states. Unlike other studies, this research did not involve recording sensor, speech, or camera data. Instead, the focus was solely on the self-reported questionnaire responses.

One intriguing avenue of exploration is the utilization of surrounding stress-related data for predicting individual stress levels. Researchers have introduced a groundbreaking approach that capitalizes on personal and social stress-related data to achieve remarkable prediction accuracy [33]. It unveils the contagious nature of stress, shedding light on how one’s focus can influence those around them. Unlike traditional stress prediction methods, which primarily rely on individual data, this study sheds light on the influence of surrounding stress, emphasizing the interconnectedness of stress levels within a social context at the workplace. In our study, by considering all measurements coming from different places, we are inherently dealing with surroundings by considering all measurements coming from different people. In this aspect, we may say that we are focusing on the well-being of the workplace rather than the individuals. In addition, their model approach encounters the time-based aspect of the dataset, likewise our study. However, in addition to stress, we explore other factors of well-being and extract the biomarkers of these factors utilizing an extensive dataset.

These studies underscore the richness and variety of approaches in stress prediction research. By leveraging different data modalities, advanced analytics, and machine-learning techniques, researchers aim to predict stress levels accurately and provide valuable insights into the intricate interplay of stress dynamics across workplaces, digital platforms, educational settings, and daily life. The cumulative efforts in this field contribute to a more profound comprehension of stress patterns and offer potential avenues for effective stress management and well-being enhancement. Building upon previous research in stress and well-being prediction utilizing wearable, mobile, ambient, and machine-learning technologies [32,33], our paper aims to address the challenges and potential areas for further investigation.

In this study, we deal with temporal patterns and dependencies within the data using methods such as RF, XGBoost, and LSTM. Based on past data, these algorithms can effectively predict stress levels, anxiety states, and variations in positive and negative affect. Importantly, these algorithms’ lagged (time-based) versions, incorporating various time windows, have shown promise in capturing nuanced temporal trends.

We have three main questions in Section 1. To the best of our knowledge, none of the well-being studies considered these four factors simultaneously. Furthermore, there is no time-based method application using the current dataset. Considering the intrinsic aspect of data collection and the nature of the states, we show that temporal dependencies have an effect. Even though RF was reported to be the most performing algorithm [32,35,36] in this dataset’s scope, we are also exploring the performance with XGBoost and LSTM alongside RF. In addition, in our previous study [36], we extracted highly related biomarkers only for the classification of the stress variable. In this study, we expand by adding three more well-being factors in a prediction context rather than classification.

Ultimately, this research aims to advance the field of time-based classification, particularly in the context of workplace well-being prediction. By addressing challenges, exploring novel methodologies, and leveraging wearable technology, we strive to contribute to developing intelligent systems that can effectively analyze and interpret temporal data for personalized applications in the workplace.

## 3. Background and Methodology for Well-Being Prediction

### 3.1. Motivation

Time-based approaches can be used in well-being detection and management by leveraging physiological and contextual data collected over time. The data streams provide rich information that can be leveraged to develop accurate and personalized stress, anxiety, and mood prediction models.

Considering the mentioned research questions in Section 1, we aim to answer the effect of time-based algorithms compared to their conventional versions and extract the related biomarkers (RQ1 and RQ3). In prediction tasks, the data format can significantly impact the modeling approach and the insights gained. Two primary data formats, time-based and conventional predictions, offer distinct characteristics and utility.

Time-series data are organized chronologically, where each observation is tied to a specific timestamp. This format introduces a crucial temporal dimension, capturing how the studied phenomenon evolves. Notable characteristics include the temporal sequencing of data points, time lags and dependencies necessitating feature selection, and the option to aggregate data over different time intervals. These features allow for modeling temporal patterns and dependencies, making time-based prediction particularly valuable in scenarios where understanding how variables change over time is essential, such as stock price forecasting, weather prediction, or user behavior analysis.

Conversely, the usual prediction data format lacks an inherent temporal component, treating each observation as independent and disregarding the order of data points. Key characteristics include the independence of observations, a focus on feature engineering that primarily considers static attributes, and the use of cross-sectional analysis, which provides a snapshot of the data at a particular time. This format is for scenarios where the sequence of observations is irrelevant or temporal aspects hold no significance in the predictive modeling process. It is often employed in areas like classification tasks and static attribute-based predictions.

### 3.2. Definitions of the Utilized Well-Being Factors

In this study, we are concentrating on four core factors of well-being: stress, anxiety, and positive and negative affect. To be more concrete about the background of these factors, we provide descriptions in the following:

#### 3.2.1. Stress

Stress is a psychological and physiological response to challenging situations or perceived threats. It often includes feelings of tension, pressure, or unease and can manifest as both mental and physical symptoms. Stress is a natural response to various life events, but excessive or chronic stress can have negative effects on one’s mental and physical well-being. As stress is a complex response, it can be influenced by a wide range of factors such as environmental (work, living conditions), psycho-social (life events, everyday challenges, lack of social relationships), biological (genetics, physical health, neurobiology), psychological (personality traits such as perfectionism, cognitive factors, resilience), lifestyle (diet and nutrition, physical activity, sleep patterns, substance use) and cultural-societal (beliefs-expectations, discrimination, social Inequality) factors.

#### 3.2.2. Anxiety

Anxiety is a persistent state of excessive worry, fear, or apprehension about future events or situations. It is characterized by heightened alertness and a sense of unease. Anxiety can be a normal response to stressors, but when it becomes overwhelming, intrusive, or impairs daily functioning, it may indicate an anxiety disorder. Anxiety disorders include various conditions, such as generalized anxiety disorder, social anxiety disorder, and panic disorder.

#### 3.2.3. Positive Affect

Positive affect refers to the experience of positive emotions, such as joy, happiness, enthusiasm, and contentment. Understanding and measuring positive affect is essential for assessing well-being and mental health. It represents the spectrum of pleasant feelings and moods that individuals may encounter in their daily lives. Positive affect is pivotal in enhancing one’s emotional state, contributing to an overall sense of happiness, and promoting psychological resilience.

#### 3.2.4. Negative Affect

Negative affect refers to the experience of negative emotions, such as sadness, anger, fear, and disgust. It encompasses a range of distressing feelings and emotional states that can impact one’s mood and overall well-being. Understanding and measuring negative affect is essential for assessing emotional health and psychological distress. Negative affect can be a normal response to adverse events, but persistent or severe negative affect may indicate underlying emotional or mental health issues.

In modern society, concerns surrounding stress and anxiety have gained prominence, impacting individuals’ well-being, productivity, and overall quality of life. Detecting and managing these conditions have become increasingly crucial, leading to a growing interest in utilizing time-based classification techniques in conjunction with wearable devices.

### 3.3. Dataset

Tesserae Dataset “https://tesserae.nd.edu/ (accessed on 31 October 2023)” [15] is used in this work. It is gathered to track office workers’ psychological and physical qualities over a year to determine their performance at work. It includes information from 757 individuals. Both a smartphone and a Garmin watch are utilized to gather data.

An activity tracking app was used to collect data from the phones. Throughout the investigation, each participant had a Garmin watch. Its battery life is five to seven days. Additionally, Bluetooth beacons have been utilized to gather details about the location, including home or work. They also gathered verbal data from social networking sites like Facebook and LinkedIn, which were inaccessible to us, as a supplement to the non-verbal measurements made by the equipment.

The data-collection campaign has been approved as a research project by the University of Notre Dame, and participants have been asked to sign a consent form. The participants were given a variety of questionnaires to complete to gather real-world data. These questionnaires include stress, exercise, sleep, mood, IQ, and job performance. Participants completed all the survey questions for each kind at the start of the trial. It is mentioned that it takes around an hour to complete. Additionally, there are daily survey questions that provide an overview of each measurement type, where the used well-being factors within the scope of this study are indicated with a star sign. The overall score of each questionnaire corresponds to one column in the daily survey scores file. Thus, filling it every day only takes a few minutes. In addition, they gathered data with a questionnaire and a follow-up survey. They were inaccessible to us, however. As a result, the questionnaires are divided into the following four sections: initial ground truth, daily surveys, exit surveys, and follow-up surveys. The exit battery and the follow-up survey were not provided to us, yet their parameter space has been given in detail in [36]. We identify the utilized ground-truth questionnaires in the scope of this study in Table 1 with a star. As we already found that personality parameters increase the recognition performance, and they were found as the most important parameters for our target variables [36], we included them in our parameter space. The rest of the surveys are utilized for the target variables, as shown in Table 1: PANAS for positive and negative affect, omnibus anxiety question for anxiety, and omnibus stress question for stress. The detailed versions of the contents of these surveys are presented in [36]. Although these data are gathered over a year, it is stated that only 56 days of daily survey data are collected “https://osf.io/yvw2f/wiki/EMAs/ (accessed on 31 October 2023)”. Thus, in the scope of this study, as we are dealing with data coming from surveys for the target variables, we are actually working on almost two months of duration of data.

### 3.4. Data Preprocessing

We conducted our analysis on a daily basis. We have features coming from the user’s activity, stress, sleep, heart rate (HR) via a Garmin watch, and phone activity via phone agents, location via Bluetooth beacons.

Even though the data were collected from 757 participants, only 727 participants’ data were available in the shared dataset. Furthermore, although it is stated that 56 days of the daily survey is collected, we observed 61 daily survey answers from some participants. The dataset was provided in separate files. To construct our dataset, we merged the required files according to *ParticipantID* and *Timestamp*. We obtained daily wearable data as input and ground-truth data as a target for each well-being factor for each participant. We considered the data from participants that only have ground-truth responses, i.e., responses to the daily surveys. After constructing the data file, we have 36,294 instances from all participants. In the final version of the data, we have 269 columns where 15 of them are the ground truth. Here, 15 columns coming from the ground truth are: *survey name*, *stress*, *anxiety*, *sleep*, *positive affect*, *negative affect*, *extraversion*, *agreeableness*, *conscientiousness*, *neuroticism*, *openness*, *total phone activity duration*, *survey sent time*, *survey start time*, *survey finish time*. We removed the unnecessary columns in our analysis *timezone change*, *local time*, *survey name*, *survey sent datetime*, *survey start datetime*, *survey end datetime* related to name, start, and end date of surveys before running factor-specific algorithms. Furthermore, we excluded other well-being factor columns while concentrating on a specific one. For example, in the case of stress, we removed *anxiety, pos affect, neg affect*.

We already reported the importance of personality attributes in stress classification in our previous work [36] and employed these parameters in our input space. These parameters include extraversion, agreeableness, conscientiousness, neuroticism, and openness. Specifically, in this dataset, these personality values are collected approximately 4–5 times during the period that we are concentrating on, i.e., two months. The selection of input columns and the construction of the final dataset is explained extensively in the “Dataset Construction Section of [36]” and a descriptive table (Table 3) is presented in that study. However, to be more clear about the features which have been used in the scope of the study, we list their names as follows: *ParticipantID*, *Timestamp*, *act in vehicle ep0*, *act in vehicle ep1*, *act in vehicle ep2*, *act in vehicle ep3*, *act in vehicle ep4*, *act on bike ep0*, *act on bike ep1*, *act on bike ep2*, *act on bike ep3*, *act on bike ep4*, *act running ep0*, *act running ep1*, *act running ep2*, *act running ep3*, *act running ep4*, *act unknown ep0*, *act unknown ep1*, *act unknown ep2*, *act unknown ep3*, *act unknown ep4*, *act walking ep0*, *act walking ep1*, *act walking ep2*, *act walking ep3*, *act walking ep4*, *active kilocalories*, *active secs*, *active time seconds*, *activity stress seconds*, *adjusted bed time*, *adjusted sleep duration*, *adjusted wakeup time*, *agreeableness*, *ave cloudcover*, *ave daytime cloudcover*, *ave daytime feelslikef*, *ave daytime heatidxf*, *ave daytime humidity*, *ave daytime pressure*, *ave daytime tempf*, *ave daytime visibility*, *ave daytime windchillf*, *ave daytime windgustmph*, *ave daytime windspeedmph*, *ave feelslikef*, *ave heatidxf*, *ave hr*, *ave humidity*, *ave pressure*, *ave stress*, *ave tempf*, *ave visibility*, *ave windchillf*, *ave windgustmph*, *ave windspeedmph*, *average hr*, *average stress level*, *bbidp median*, *calories active hrs*, *caloriesdp median*, *conscientiousness*, *date*, *distance in meters*, *duration in seconds*, *extraversion*, *floors climbed*, *garmin calories max*, *garmin calories mean*, *garmin calories median*, *garmin calories min*, *garmin calories std*, *garmin steps max*, *garmin steps mean*, *garmin steps median*, *garmin steps min*, *garmin steps std*, *gimbal active hrs*, *gimbal ep0*, *gimbal ep1*, *gimbal ep2*, *gimbal ep3*, *gimbal ep4*, *gimbaldp median*, *high stress duration seconds*, *highly active secs*, *hr active hrs*, *hrdp median*, *locdp median*, *low stress duration seconds*, *max cloudcover*, *max daytime cloudcover*, *max daytime feelslikef*, *max daytime heatidxf*, *max daytime humidity*, *max daytime pressure*, *max daytime tempf*, *max daytime visibility*, *max daytime windchillf*, *max daytime windgustmph*, *max daytime windspeedmph*, *max feelslikef*, *max heatidxf*, *max hr HR*, *max hr actSleepDailyStress*, *max humidity*, *max pressure*, *max stress*, *max stress level*, *max tempf*, *max visibility*, *max windchillf*, *max windgustmph*, *max windspeedmph*, *median hr*, *median stress*, *medium stress duration seconds*, *mildest daytime weathercode*, *mildest daytime weatherdesc*, *mildest weathercode*, *mildest weatherdesc*, *min cloudcover*, *min daytime cloudcover*, *min daytime feelslikef*, *min daytime heatidxf*, *min daytime humidity*, *min daytime pressure*, *min daytime tempf*, *min daytime visibility*, *min daytime windchillf*, *min daytime windgustmph*, *min daytime windspeedmph*, *min feelslikef*, *min heart rate*, *min heatidxf*, *min hr*, *min humidity*, *min pressure*, *min stress*, *min tempf*, *min visibility*, *min windchillf*, *min windgustmph*, *min windspeedmph*, *mode daytime weathercode*, *mode daytime weatherdesc*, *mode hr*, *mode stress*, *mode weathercode*, *mode weatherdesc*, *moderate intensity duration seconds*, *neg affect*, *neuroticism*, *num samples*, *num samples actSleepDaily*, *num samples stress*, *openness*, *quality activity*, *quality bbi*, *quality calories*, *quality gimbal*, *quality hr*, *quality steps*, *range hr*, *range stress*, *rest stress duration seconds*, *resting hr*, *severest daytime weathercode*, *severest daytime weatherdesc*, *severest weathercode*, *severest weatherdesc*, *steps*, *steps active hrs*, *stepsdp median*, *stress duration seconds*, *total activity secs*, *total daytime precipmm*, *total pa d*, *total precipmm*, *total snowcm*, *unique act count*, *unique gim count*, *unlock duration ep0*, *unlock duration ep1*, *unlock duration ep2*, *unlock duration ep3*, *unlock duration ep4*, *unlock num ep0*, *unlock num ep1*, *unlock num ep2*, *unlock num ep3*, *unlock num ep4*, *vigorous intensity duration seconds*. For more details and descriptions, please visit the project’s website “https://osf.io/yvw2f/wiki/EMAs/ (accessed on 31 October 2023)”.

We examined missing values among the independent, i.e., input, variables. Some attributes were never collected from some participants. Thus, an imputation could not be applied to these attributes. Among them, if there is a lack of many participants’ data, their removal by row leads to a high decrease in our dataset. Instead, we deleted those columns. These are *act (activity) still*, *light mean*, *garmin hr (heart rate) min*, *garmin hr max*, *garmin hr median*, *garmin hr mean*, *garmin hr std*, *ave (average) hr at work*, *ave hr at desk*, *ave hr at desk*, *ave hr not at work*, *call in num (number)*, *call in duration*, *call out num*, *call out duration*, *call miss num*, and their derivatives according to time episodes. However, when we have data columns with missing values at the person level, we compute them by applying the mean operation for each participant. Even after this operation, there were some missing values due to the lack of column values at a person level. We had to remove them row by row, resulting in the removal of around 5000 rows (from 36,284 to 31,772). During this process, we observed that missing values at the person level for one parameter were highly correlated with the presence of missing values for other parameters as well. In addition, we applied data scaling using a scale mapper.

We proceeded with the same preprocessing steps for each target factor and achieved the same parameter space. In the end, we have 194 columns with input and target parameters and around 31,700 rows due to changes in missing values in the target column. More precisely, it is 31,772 for stress, 31,731 for anxiety, 31,703 for positive affect, and 31,702 for negative affect. To give an idea about the dataset, we present some columns with their corresponding values and participant ID in Table 2 for the positive affect target variable.

### 3.5. Creation of Lagged (Time-Based) Dataset

Time-based prediction techniques combined with deep-learning architectures, ensemble techniques, and multimodal data fusion are powerful methods for temporal data. By leveraging wearables and exploring synergies with emerging technologies, the field of time-based classification is poised to make further strides in accurately analyzing and interpreting temporal data.

When dealing with time-series data in healthcare, it is essential to consider temporal dependencies and trends. The traditional version of the indicated algorithms (RF, XGBoost) may not capture these aspects effectively, as it does not inherently account for the sequential nature of time-series data. This is why we created lagged versions of the dataset. Also, even though LSTM considers the time aspect inherently, its lagged version, often referred to as Time-Lagged LSTM, extends the capabilities of traditional LSTMs by explicitly incorporating lagged features into the model. They excel at capturing complex temporal relationships and can provide valuable insights for time-series analysis in healthcare.

A lagged version introduces the concept of time lags to consider the time aspect in the data. It involves the creation of lagged versions of the features, where each data point is associated with previous time steps to incorporate historical information. This approach can significantly enhance the predictive performance, especially when dealing with time-series data. The model better understands how data evolves over time, making it a valuable tool for time-sensitive healthcare applications.

To explain this concept, consider an employee with a certain level of well-being (*W*) in terms of stress, anxiety, positive and negative affect separately (either one of them depending on the scenario) during a certain period t are the following:

(1) $$W\left(t\right)=\left[w_{t-n},w_{t-(n-1)},\ldots ,w_{t-1},w_{t}\right]$$

where $W_{t}$ represents the well-being parameter value *W* over a period t, on a range of 1 to 5 for stress and anxiety, 5 to 25 for positive and negative affect. $w_{t-n},w_{t-(n-1)},\ldots ,w_{t-1},w_{t}$ are the individual measurements of the well-being parameter *W* at different time points within the time window. Therefore, $w_{t-n}$∈[1,…,5] or [5,…,25] depending on the scenario of well-being, and *n* is the size of the time window, indicating the number of past measurements considered in the measurements of well-being parameters. In this study, we employed 1,3,7,15,30 as look-back (n) sizes to consider daily, weekly, fortnightly, and monthly results. Additionally, we wanted to observe the change between daily and weekly performance, so we also employed a 3 day look-back roughly to state half of the week. We could have considered longer periods, such as yearly and seasonal periods. However, as we have only 56 days of data, these approaches are not applicable due to the dataset constraints.

### 3.6. Prediction Algorithms

#### 3.6.1. Random Forest

RF is an ensemble learning method that combines multiple decision trees to make more accurate predictions or classifications. It is particularly effective for various tasks, including regression and classification. It works by creating a multitude of decision trees during training. Each tree is constructed based on a random subset of the dataset (bootstrapping), and at each node, a random subset of features is considered. This randomness helps in reducing overfitting and improving generalization. During prediction, each tree provides an output (class label or numerical value), and the final prediction is determined by averaging (for regression) or taking a majority vote (for classification) of these individual tree outputs.

#### 3.6.2. XGBoost

XGBoost, short for Extreme Gradient Boosting, is another robust machine-learning algorithm commonly used in various domains, including healthcare. Again, it is an ensemble learning technique that combines the predictions of multiple decision trees to achieve high predictive accuracy. XGBoost is known for its efficiency, scalability, and ability to handle various data types, making it a popular choice for predictive modeling tasks.

In the healthcare domain, XGBoost can be used for disease diagnosis, patient risk stratification, and medical image analysis. It excels in capturing complex patterns and relationships within healthcare data.

#### 3.6.3. Long Short-Term Memory

LSTM is a recurrent neural network (RNN) architecture that handles sequential data. It is well-suited for time-series analysis, natural language processing, and other applications. LSTMs are known for their ability to capture long-range dependencies in sequences while avoiding the vanishing gradient problem, which can hinder the training of traditional RNNs. LSTM models can be used in healthcare for patient monitoring, disease prediction, medical image analysis, and more. They excel in scenarios where the order and temporal relationships between data points are critical.

## 4. Performance of Well-Being Factors Prediction

### 4.1. Implementation Details

We employed traditional RF, XGBoost, LSTM, and their lagged versions as prediction/regression models. We used the Random Forest (RF) algorithm in our analyses because it is an ensemble method and performs better among the other used methods in the literature in this domain [4], XGBoost as it is indicated to perform better compared to deep-learning techniques and even better once combined with deep-learning compared to alone version [37], and LSTM as it considers time aspects of data inherently. We also have their lagged versions for 1,3,7,15,30 days look-back to consider the contribution of past data points and 1,3,7 days lookup to understand up to how many days we can reach reasonable errors and which one’s performance is better.

To be fair, in comparisons, as the parameter space is 99% is similar (in Section 3.4, the total number of instances per target value was given), and only the target column changes for each scenario specific to different well-being targets. We employed the same preprocessing steps for the dataset and hyper-parameter values for the applied algorithms. After hyper-parameter optimization, we found that in scikit-learn n estimators 1000, criterion gini, min sample split 2, min samples leaf 1, max features sqrt combination reveal the best performance for RF, 50 layered LSTM with epochs 50, batch size 72, adam optimizer with learning rate 0.001, hyperbolic tangent as an activation function and MAE as a loss function, and for XGBoost colsample bytree 0.8482, gamma 4.34, max depth 3, min child weight 7.0, reg alpha 107.0, reg lambda 0.8336, n estimators 20.

We employed 80% and 20% train and test dataset sizes, respectively, as in [36]. However, in this study, we also considered a person’s ID information for splitting even though it is not personalized yet. When we split the dataset sharply to obtain 80% and 20%, it corresponded to splitting one person’s data, some parts in the training set and some in the test. To solve this issue, we assigned 443 person’s data in the training set and 100 person’s data in the test set to reach roughly 80% and 20% splitting. In the context of neural networks, it is a common practice to have three different sets: train, test, and validation. Here, we did not employ a three-set division. The decision to exclude a separate test set was motivated by two key factors. First, training was stopped when no improvement between epochs was observed. This practice aligns with the common approach to halt training when the validation metric ceases to improve, indicating potential diminishing returns and the risk of overfitting. Second, the availability of a large and diverse dataset played a significant role in this decision. A substantial dataset can mitigate overfitting risks by providing a robust measure of the model’s generalization capability without the need for a dedicated test set, which was not observed during our experiments.

The experiments are performed on macOS, a 2.7 GHz quad-core Intel Core i7 processor with 16 GB of 2133 MHz LPDDR3 SDRAM using the Google Colab platform and Python 3.10 scripts.

### 4.2. Biomarkers of Well-Being Factors

**Figure 1:**
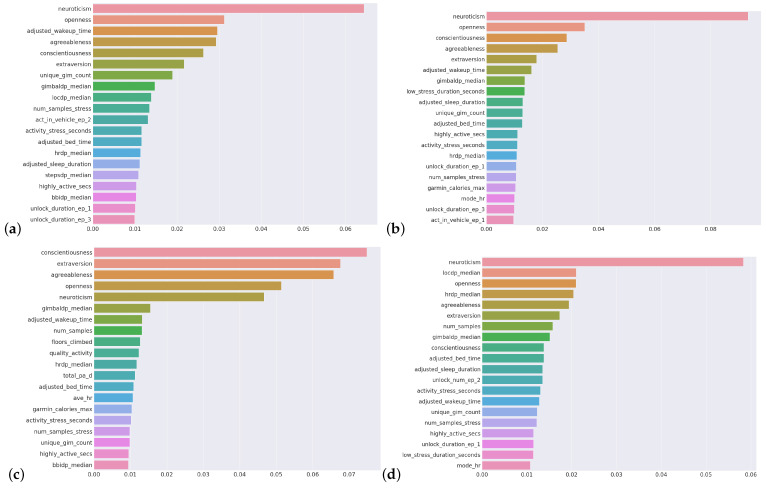
The most important features per target (**a**) Stress (**b**) Anxiety (**c**) Positive Affect (**d**) Negative Affect.

Before examining the performance of time-based versus non-time-based predictions, we extracted the most effective parameters, i.e., biomarkers, on the target well-being factors. We employed Random Forest for feature importance. For each target variable, top 20 features are presented in Figure 1; corresponding subfigures are as follows: for stress (a), for anxiety (b), for positive affect (c) and negative affect (d). As these explain the health-related components, in the literature, they are called digital biomarkers [38]. By doing this, we aim to answer RQ1, mentioned in Section 1. In our previous study [36], concentrating on a stress classification task, we have already found that stress has two overwhelming biomarkers: anxiety and negative affection. This was also the case in this prediction study. As these two factors are also our target variables in the scope of this work, we excluded the other examined factors while targeting one from the set of well-being factors. That is why they are not listed in the most important biomarkers lists.

Similar to the results presented in the stress classification study in [36], the personality factors, which are extraversion, agreeableness, conscientiousness, neuroticism, and openness, are all found among the most important factors, almost always in the top 5 again (Figure 1) in this study, for all target variables in a separate manner for a regression problem. As well as the personality attributes, we notice that some others, such as sleep-related parameters, including bedtime and wakeup time, are the common attributes in all factors. Similarly, the number of stress samples, highly active seconds, and activity stress seconds are also common parameters. In addition, phone unlock duration, which comes from phone activity, is common in three target factors, i.e., stress, anxiety, and negative affect.

As we are dealing with multiple types of inputs, i.e., biomarkers, coming from different modalities, i.e., measurements from different devices (phone, smartwatch) and personality surveys, we wanted to understand the importance of the modalities for future studies to decide on what types of data should be collected to predict the well-being factors. The modalities are phone activity, personality surveys, and the ones coming from the Garmin watch: sleep, stress, daily (this includes a summary of daily recordings, such as distance in meters), activity, and heart rate.

We rank the biomarkers according to their corresponding modality set. Since we have extracted the most important 20 features, rankings are from 20 to 1, 20 as the most important one. We sum the rankings of the biomarkers when computing the importance of the modality they belong to. For example, bedtime, sleep duration, and wakeup time biomarkers are under the sleep modality from the watch. If these were found among the most important 20 features, we sum their ranking values for the sleep modality.

**Figure 2:**
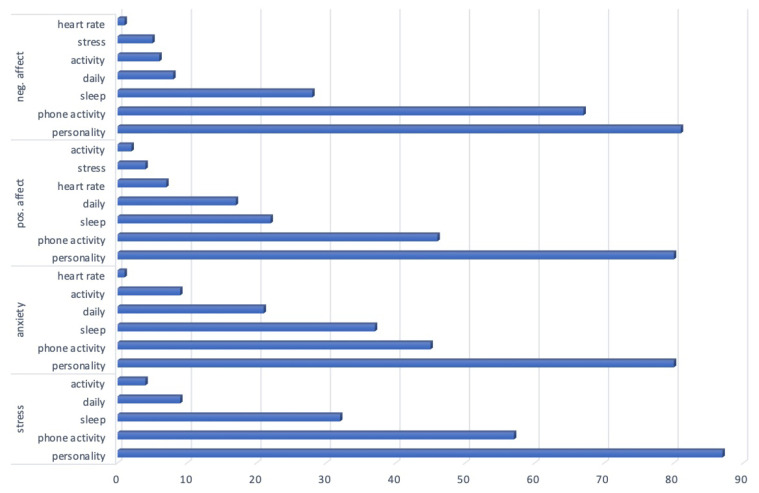
Modality rankings.

In Figure 2, we provide the rankings of the biomarkers according to their modalities from the phone, the wearable watch (Garmin), and surveys (personality). We observed a similar ranking as *personality*, *phone activity*, *sleep*, *daily activity* for each target factor. The rest of the rankings are as follows *activity* for stress; *activity*, *heart rate* for anxiety; *heart rate, stress, activity* for positive affect; *activity*, *stress*, *heart rate* for negative affect (Figure 2). Even though we are dealing with a regression problem in this study, we observe a similar modality ranking as stress classification with resolved class imbalance cases [36]. In addition, this ranking is the same for each target variable, which shows the transitivity of these modalities across targets.

As well as wearable and phone devices’ various measurements to relate to the psychology literature in answering our second research question (RQ2), we focus on the parameters coming from sleep, stress, heart rate, and activity modalities. Please note that contrary to target variables from daily survey data, these parameters come from the device measurements. This is why we encounter stress sample numbers among the important features for stress targets. Observing these parameters on the parameter space validates device measurements with the subjective values.

It is observed that sleep and activity occur commonly among all target important variables. In the literature, it is stated that there is a strong relationship between sleep and stress [39,40,41], sleep and anxiety [23,42,43], sleep and mood (positive and negative affect) [44]. In addition, it is found that sleep patterns are among the most important health-related factors [45]. Our study also validates these results (Figure 2) by having sleep in the upper ranking of importance compared to activity, stress, and heart rate for each one of the target variables.

In [24,46], researchers found that there is a relation between physical activity, anxiety, and stress. It is stated that with the increase of physical activity levels, there is less subsequent stress and negative affect, as well as more positive affect [47]. In our results, activity-related parameters are found towards the end of the importance rankings in Figure 1, yet the results shown are the most important among the whole parameter space. Thus, again, the results aligned with the psychological literature.

Interestingly, most of the stress-related factors were found to be important for positive and negative affect. In [48,49], it is reported that perceived stress level is associated with positive and negative affects’ reactivity to current events while trait anxiety moderated reactivity of agitation. Also, it is stated that the intensity and duration are important for their relationship. This finding also overlaps with our findings.

In conclusion, with this study, we provide the most important physiological parameters that align with the existing studies in the literature from the psychological perspective.

### 4.3. Conventional vs. Time-Based Prediction Performances

In well-being, four key factors available in this dataset provide insights into an individual’s psychological state. Stress and anxiety, rated on a scale from 1 to 5, encompass the emotional responses to challenges and worries. Stress levels indicate the intensity of pressures and challenges experienced, while anxiety reflects the degree of unease and fear. On the positive side, positive affect, rated from 5 to 25, measures the abundance of positive emotions, indicating the extent of happiness and enthusiasm. Conversely, negative affect, also rated from 5 to 25, gauges the presence of negative emotions, portraying the degree of distress and unhappiness. These measures collectively offer a comprehensive view of an individual’s emotional state and overall well-being, from coping with stress and anxiety to experiencing positive and negative emotions.

**Figure 3:**
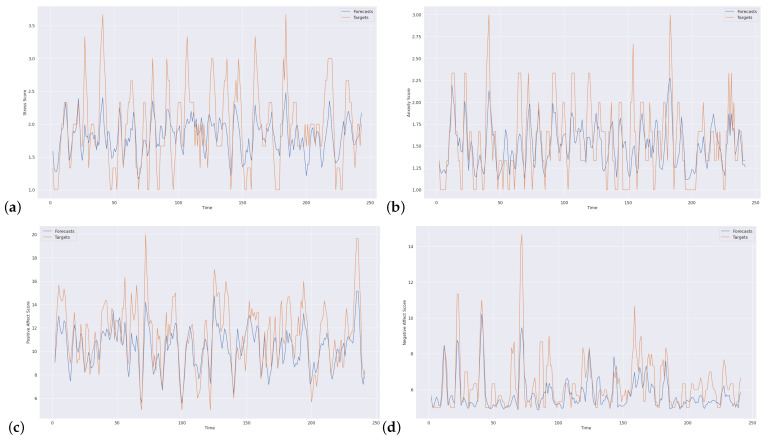
Forecast visuals with XGBoost 7 days prior (**a**) Stress (**b**) Anxiety (**c**) Positive Affect (**d**) Negative Affect.

As stated earlier, we are using RF, XGBoost, and LSTM algorithms and their lagged variations in this study. This section will refer to them as conventional and time-based versions to facilitate the understanding. The performances of the algorithms are measured in terms of mean absolute error (MAE) to be able to account for negative errors properly. The reason behind the choice of MAE comes from the observation of the model’s under-prediction, as one may observe in Figure 3. Thus, no sign difference suits the usage of MAE as an error metric. The X-axis in Figure 3 represents the index ranging from 0 to 249, corresponding to the data points in the lagged dataset. Since the dataset is constructed with consideration for the time aspect, these indexes are also related to time.

The results are presented in Table 3. When we look at the results of the conventional versions of the algorithms, we see that RF and XGBoost results are closer, but LSTM is better since it captures the time aspect in the data intriguingly. On the other hand, we observed that XGBoost performs far better in terms of running time compared to RF. RF is a versatile algorithm often used in predictive modeling tasks. We used RF to predict outcomes based on historical data. However, RF may not capture temporal dependencies and patterns as effectively as time-based models. It performed reasonably well but did not outperform other models in capturing time-related factors. LSTM is a deep-learning model designed for sequential data. We applied LSTM to capture intricate time dependencies, which can be highly effective for time-series forecasting.

In lagged (time-based) versions of data, we considered different time windows (1 day, 3 days, 7 days, 15 days, and 30 days look-back) and allowed for lookahead predictions (1 day, 3 days, and 7 days lookup) involved incorporating historical data. However, in order not to present an overwhelming table, we provided only some combinations of look-back and lookup parameter space.

When we compare conventional and time-based versions, we observe a clear improvement in the performances with the time-based version regardless of the algorithm. The best-performing configuration for time-based versions belongs to LSTM. Particularly, the 3-day look-back and 1-day lookup likely excelled because the combination effectively captures recent trends while considering the influence of the previous days. This configuration balanced short-term adaptability with longer-term patterns, providing the most accurate results for almost all target variables. We observed better results with a 15-day look-back only for the stress target. We interpret it as the intriguing aspect of the data; it may represent the stressful period of work while collecting the dataset. However, we are not fully sure about this since we do not have ground-truth recordings for this issue, or in other words, the daily lives of the participants.

Even though LSTM was found to be the best-performing one, its results are very close to XGBoost, and its results are even better in some cases (e.g., 15 look-back-1 lookup). However, XGBoost’s execution time is shorter than LSTMs in all cases. Furthermore, the data ranges for positive and negative affect are different and wider compared to stress and anxiety. We observe higher error values for them.

To the best of our knowledge, this is the first study that employs different well-being factors at the same time and also focuses on their time-based results in the scope of a prediction problem. Nevertheless, to compare our findings with the literature where time-based techniques were utilized in other well-being datasets [33], we see that they employed daily and weekly resolutions. They employed different models such as LogR, DT, and ADA on different kinds of features, yet they also concluded an increase in performances from daily to weekly transition. More precisely, for the stress prediction study case, they achieved a nearly 59% and 56% F-score on their data and the StudentLife dataset correspondingly. These performances increased to 72% with their data and 61% in the case of StudentLife. In another study [35], where the same dataset is used, the authors focused on creating a benchmark for predictive analysis by integrating several aspects of an individual’s physical and psychological behavior, psychological states and traits, and job performance. They employed High Order Networks to include the time aspect of the data while combining the modalities. Although our study deals exclusively with well-being factors, this study may also provide insights into higher-level components. Additionally, in [36], we focused solely on the stress variable and applied conventional techniques. However, as we were dealing with a classification problem in that study, it would not be appropriate to compare its results with the regression results found in the scope of this study.

To show the differences between predictions (forecasts) and ground-truth target values, we present detailed prediction visualization for XGBoost for 7 days look-back predictions in detail in Figure 3 for stress, anxiety, positive affect, and negative affect. In these figures, the commonly observed pattern is that the predicted values are lower than the original values. Thus, models under-predict the values, but the patterns are being captured.

In conclusion, we found that time-based versions of the applied algorithms perform better than conventional ones in this study’s scope, which answers our third question (RQ3). The best-performing one is LSTM, with a 3-day look-back and a 1-day lookup for almost all target variables.

Please note that the results presented in our study are based on predictions made using a test set that includes data from individual participants. However, it is important to clarify that our analysis and research objectives are primarily focused on deriving aggregate or collective insights rather than providing personalized predictions. The distinction lies in the way we interpret and utilize the results. Although our test set does consist of data from individuals, our primary aim is to derive overarching patterns and trends in the data that can be applied at a higher level. In essence, we are looking for trends or behaviors that are consistent across different people rather than focusing on precise individual-level predictions. This approach enables us to draw broader conclusions about the dataset as a whole and assess the model’s effectiveness in capturing general trends in the data. Although individual-level predictions are indeed valuable in further contexts, our current study is designed to contribute insights into collective behaviors and trends related to the predictive task at hand.

## 5. Conclusions

We focused on the prediction of well-being factors, namely stress, anxiety, and positive and negative affect, using the Tesserae dataset collected from office workers. We answered three research questions related to digital biomarkers affecting these factors (RQ1), their alignment with the conventional psychology literature (RQ2), and time-based performances of applied methods (RQ3).

We found similar modality rankings for each target variable. Even though we are working on prediction problems rather than classification, these findings align with the one-factor stress classification results after imputing missing values [36].

In addition, we validated our findings by examining the conventional psychology literature where there is no wearable device usage. We found that device-measured modalities aligned with the paper-based studies. Furthermore, we found that we achieve better prediction performances when we consider the time aspect of the data. In particular, LSTM performs the best compared to other ensemble algorithms.

Our study’s findings reveal the potential for these models to play a significant role in various practical applications. These include early interventions and support to monitor and address periods of heightened stress and anxiety. Moreover, these models could facilitate continuous mental health monitoring through wearable devices, offering individuals valuable insights into their well-being. In workplace settings, integrating these models into well-being programs can help manage stress and anxiety levels among employees, contributing to healthier and more productive work environments. At a broader level, our models offer data-driven insights that can inform public health initiatives, educational programs, and policy decisions geared toward addressing mental health challenges. However, it is crucial to emphasize that implementing these models in real-world scenarios requires thorough validation, ethical considerations, and careful integration. Additionally, a more in-depth discussion on the limitations, challenges, and ethical aspects of applying predictive models to mental health prediction would be a valuable complement to our study.

There are also limitations of this study that we want to enhance in further studies. We are constantly working on the available data points by excluding missing ones. Since this amount is vast, their imputation might help to improve precision and may lead to better performances. Also, even though we considered person-based splitting during the train-test split, we did not consider a personalized approach during the analysis. In the future, we are planning to integrate the personalization aspect into the analysis.

## Figures and Tables

**Table 1:** Details of the daily questionnaires (* correspond to the ones used in this study).

Questionnaires	Content
Daily Surveys	Big Five Inventory (BFI) *: Extraversion, Agreeableness, Conscientiousness, Neuroticism, Openness, Positive and Negative Affect Schedule (PANAS) *, Omnibus Anxiety Question *, MITRE Omnibus Stress Question *, MITRE Physical Activity Assessment MITRE Sleep Assessment

**Table 2:** A snapshot of the data for positive affect.

ID	Timestamp	Active Secs	…	Resting Hr	Steps	Stress Duration Seconds	…	Positive Affect
4188a14d4edd8eb1cac1a146d9f88aee	21 February 2018	2417	…	57.0	6634	34,560.0	…	14
4188a14d4edd8eb1cac1a146d9f88aee	22 February 2018	2557	…	55.0	6008	21,960.0	…	14
4188a14d4edd8eb1cac1a146d9f88aee	23 February 2018	5849	…	55.0	9008	26,880.0	…	15
…	…	…	…	…	…	…	…	…
4188a14d4edd8eb1cac1a146d9f88aee	27 April 2018	3595	…	72.0	4837	12,240.0	…	15
…	…	…	…	…	…	…	…	
c30291318f6d680bd65666c183f6bb5e	11 January 2018	4988	…	57.0	10,550	19,020.0	…	11
…	…	…	…	…	…	…	…	…
c30291318f6d680bd65666c183f6bb5e	13 March 2018	5109	…	50.0	11,002	46,140.0	…	9

**Table 3:** Prediction performances in terms of MAE (best results are shown as bold).

Type	Method	Stress	Anxiety	Positive Affect	Negative Affect
Conventional	Random Forest (RF)	0.6702	0.6508	3.2955	2.2088
	XGBoost	0.6910	0.6316	3.4269	1.7874
	LSTM	0.6443	0.6445	3.2325	1.5121
Time-Based	RF (1 day look back-1 day lookup)	0.5922	0.4700	2.4881	1.4999
	XGBoost (1 day look back-1 day lookup)	0.6319	0.4745	2.4947	1.4304
	LSTM (1 day look back-1 day lookup)	0.5377	0.3897	2.5075	1.3841
	RF (3 days look back-1 day lookup)	0.5907	0.4628	2.2741	1.3118
	XGBoost (3 days look back-1 day lookup)	0.5952	0.4653	2.1851	1.2466
	LSTM (3 days look back-1 day lookup)	0.5394	**0.3729**	**2.1387**	**1.0574**
	RF (7 days look back-1 day lookup)	0.5808	0.4575	2.3130	1.2717
	XGBoost (7 days look back-1 day lookup)	0.5791	0.4527	2.2623	1.0866
	LSTM (7 days look back-1 day lookup)	0.5111	0.3846	2.1923	**1.0522**
	XGBoost (15 days look back-1 day lookup)	0.6327	0.5736	2.4947	1.2982
	LSTM (15 days look back-1 day lookup)	**0.4718**	0.4752	2.3795	1.3159
	XGBoost (30 days look back-1 day lookup)	0.6518	0.6025	2.1583	1.3630
	LSTM (30 days look back-1 day lookup)	0.5004	0.4804	2.3929	1.4011
	RF (3 days look back-3 days lookup)	0.5950	0.5239	2.5205	1.3992
	XGBoost (3 days look back-3 days lookup)	0.6078	0.5356	2.3898	1.3983
	LSTM (3 days look back-3 days lookup)	0.5547	0.4600	2.3425	1.2564
	RF (7 days look back-3 days lookup)	0.6324	0.5605	2.6482	1.4781
	XGBoost (7 days look back-3 days lookup)	0.6122	0.5386	2.5328	1.4341
	LSTM (7 days look back-3 days lookup)	0.5480	0.4805	2.6318	1.2277
	XGBoost (15 days look back-3 days lookup)	0.6371	0.5754	2.3931	1.5495
	LSTM (15 days look back-3 days lookup)	0.5470	0.4931	2.6475	1.3904
	XGBoost (30 days look back-3 days lookup)	0.6542	0.6067	2.4716	1.6104
	LSTM (30 days look back-3 days lookup)	0.5576	0.5046	2.8832	1.3621
	XGBoost (7 days look back-7 days lookup)	0.6203	0.5450	2.5584	1.4378
	LSTM (7 days look back-7 days lookup)	0.5947	0.5023	2.6342	1.3411
	XGBoost (15 days look back-7 days lookup)	0.6396	0.5846	2.5653	1.5649
	LSTM (15 days look back-7 days lookup)	0.6018	0.5322	2.8423	1.5316
	XGBoost (30 days look back-7 days lookup)	0.6548	0.6125	2.5487	1.6158
	LSTM (30 days look back-7 days lookup)	0.5802	0.5106	2.9486	1.4353

## Data Availability

Tesserae dataset that support the finding of this study are available upon data usage agreement at https://tesserae.nd.edu/ accessed on 4 October 2023.
